# Biomarkers of lymph node metastasis in colorectal cancer: update

**DOI:** 10.3389/fonc.2024.1409627

**Published:** 2024-09-12

**Authors:** Xiao Zhu, Shui-quan Lin, Jun Xie, Li-hui Wang, Li-juan Zhang, Ling-ling Xu, Jian-guang Xu, Yang-bo Lv

**Affiliations:** ^1^ Department of Colorectal Surgery, The Quzhou Affiliated Hospital of Wenzhou Medical University, Quzhou People’s Hospital, Quzhou, China; ^2^ Department of Endocrinology and Metabolism, Affiliated Hospital of Integrated Traditional Chinese and Western Medicine, Nanjing University of Chinese Medicine, Nanjing, China; ^3^ Department of Gastroenterology, The Quzhou Affiliated Hospital of Wenzhou Medical University, Quzhou People’s Hospital, Quzhou, China

**Keywords:** colorectal cancer, lymph node metastasis, biomarker, lymphatic invasion, mechanisms, pathophysiology

## Abstract

Colorectal cancer (CRC) ranks as the second leading cause of cancer-related deaths globally, trailing only behind lung cancer, and stands as the third most prevalent malignant tumor, following lung and breast cancers. The primary cause of mortality in colorectal cancer (CRC) stems from distant metastasis. Among the various routes of metastasis in CRC, lymph node metastasis predominates, serving as a pivotal factor in both prognostication and treatment decisions for patients. This intricate cascade of events involves multifaceted molecular mechanisms, highlighting the complexity underlying lymph node metastasis in CRC. The cytokines or proteins involved in lymph node metastasis may represent the most promising lymph node metastasis markers for clinical use. In this review, we aim to consolidate the current understanding of the mechanisms and pathophysiology underlying lymph node metastasis in colorectal cancer (CRC), drawing upon insights from the most recent literatures. We also provide an overview of the latest advancements in comprehending the molecular underpinnings of lymph node metastasis in CRC, along with the potential of innovative targeted therapies. These advancements hold promise for enhancing the prognosis of CRC patients by addressing the challenges posed by lymph node metastasis.

## Introduction

Colorectal cancer (CRC) is one of the three most common cancers and the second leading cancer killer worldwide (1). The global incidence of colorectal cancer (CRC) has been steadily increasing, with an annual rise of 3.2%. In 2020 alone, an estimated 1.93 million new cases of CRC were diagnosed, leading to approximately 0.94 million deaths worldwide. Projections suggest that by 2040, the number of new CRC cases globally is expected to escalate to 3.2 million (2). China and the United States have the highest estimated number of new CRC cases in the next 20 years (2). China is anticipated to have a 64% increase in colorectal cancer cases from 0.56 million in 2020 to 0.91 million in 2040 (2). The escalating prevalence of colorectal cancer (CRC) represents an increasingly pressing global public health concern. This disease manifests as a complex and molecularly diverse entity, exhibiting distinct genomic profiles across various subtypes. Such heterogeneity not only impacts prognosis but also shapes the response to therapeutic interventions (3). The identification and validation of novel patient stratification methods for colorectal cancer (CRC), coupled with the exploration of potential therapeutic targets, and advancements in diagnostic approaches, hold promise for enhancing treatment outcomes in CRC (4). The main cause for the high mortality is metastases in CRC patients before or after curative treatment (5).

Lymph node metastasis is considered a pivotal determinant for forecasting disease recurrence and survival rates among individuals diagnosed with colorectal cancer (6). The assessment of lymph node status through examination of the resected specimen serves as a cornerstone of the current staging system for colorectal cancer. Moreover, it plays a pivotal role in determining the necessity of adjuvant chemotherapy following surgical resection (6). The rate of lymph node metastasis (LNM) in early CRC is 6.9%–19.6% (7). The incidence of lymph node metastasis (LNM) in colorectal carcinoma (CRC) with a submucosal (SM) invasion depth of 1,000 µm or more can reach up to 12.5%, making it the primary indication for additional resection in routine clinical practice (7). Preoperative assessment of lymph node metastasis (LNM) risk in colon cancer serves two crucial purposes: first, it furnishes vital prognostic information; second, it aids in devising optimal therapeutic and staging strategies, especially in the context of neo-adjuvant treatment approaches. While the precise mechanism remains elusive, it is theorized that tumor cells disseminate from the primary tumor site to regional lymph nodes through lymphatic vessels, subsequently progressing to distant organs. As such, regional lymph node metastasis is considered a pivotal step in the dissemination of tumor cells in colorectal cancer (6).

Lymph node metastasis stands as a pivotal determinant of colorectal cancer (CRC) staging. Its presence signifies an advanced disease stage, carrying a heightened risk of recurrence and a poorer prognosis. Integral to tumor staging systems like the tumor, node, metastasis (TNM) classification, lymph node involvement significantly informs treatment strategies and prognostic evaluations (8). In CRC, lymph node metastasis serves as a robust predictor of disease recurrence. Patients with lymph node-positive disease face an elevated risk of tumor recurrence, both locally within the pelvic region and at distant sites. Consequently, the detection of lymph node metastasis influences decisions regarding adjuvant chemotherapy, owing to its indication of a higher propensity for systemic disease spread. Lymph node status plays a decisive role in tailoring treatment approaches for CRC patients (9). While individuals with lymph-node-negative disease (stage I and II CRC) may qualify for curative surgery alone, those with lymph-node-positive disease (stage III CRC) typically undergo adjuvant chemotherapy post-surgery to mitigate recurrence risks. Additionally, neoadjuvant chemotherapy may be considered for patients with locally advanced disease, aiming to downstage tumors and facilitate surgical resection.

Furthermore, lymph node status emerges as a potent prognostic factor in CRC, with lymph-node-negative patients generally exhibiting better outcomes and higher survival rates compared to their lymph-node-positive counterparts. Within the lymph-node-positive population, the number of involved lymph nodes (i.e., the lymph node ratio) and the presence of extranodal extension further refine prognostic stratification.

The extent of lymphadenectomy during CRC surgery is intricately linked to the presence of lymph node metastasis. Adequate lymphadenectomy is imperative for precise staging and prognostication, and for optimizing oncologic outcomes. Moreover, the thorough examination of lymph nodes (lymph node yield) serves as a vital quality indicator in CRC surgery, influencing staging accuracy and guiding treatment decisions. In this study, we comprehensively review recent findings regarding biomarkers associated with lymph node metastasis in colorectal cancer. Drawing upon the latest advancements, we analyze their specific roles in the process of lymph node metastasis in colorectal cancer. This critical evaluation offers novel insights aimed at enhancing our understanding of the underlying molecular mechanisms involved in colorectal cancer lymph node metastasis and holds promise for the development of targeted therapeutics aimed at inhibiting this metastatic progression.

## Pathophysiology of lymph node metastases in colorectal cancer

Lymphatic metastasis can occur in most epithelial-derived malignant tumors in the early stage, which is related to the special structure of lymphatic vessels (10). Lymphatic vessels are different from blood vessels. The lumen of lymphatic capillaries is relatively large and irregular, and the walls are thin. They are only composed of a single layer of endothelial cells and extremely thin connective tissue (11). The walls of the lymphatic capillaries lack an intact basement membrane and pericytes, but there is a layer of cribriform stroma outside the endothelium (12). This layer of matrix mainly contains type IV collagen and a small amount of laminin, almost no heparin sulfate proteoglycan and fibronectin (13). When the tissue becomes cancerous, the local expression of matrix metalloproteinases is increased to degrade the extra-endothelial matrix (14). Tumor cells adhere to the exposed fibronectin outside the endothelium and are prone to infiltration and metastasis. Another study also confirmed that the affinity of cancer cells to the basement membrane defect site was significantly higher than that of the basement membrane intact site (15). There are also “anchor wires” around the lymphatic capillaries to connect the vessel wall and the surrounding connective tissue. Under physiological conditions, the flow of lymph depends on the perivascular sheath, the anchor wire, and the contraction of the surrounding elastic connective tissue (16, 17). When cancer cells invade, the permeability of lymphatic capillaries increases, and the increase in exudate causes edema and pressure of perivascular tissues (17). After stimulating the anchor filaments, they relax and open the lumen, and the overlapping connections between endothelial cells also open, which is undoubtedly the entry of tumor cells. Lymphatic vessels provide efficient passage.

Lymph node metastases in colorectal cancer occur through a complex interplay of cellular and molecular mechanisms. Here is a simplified overview of the pathophysiology involved: 1) initiation of primary tumor: colorectal cancer typically originates from benign adenomatous polyps in the colon or rectum (18). The transformation of normal colonic epithelial cells into malignant cells is driven by genetic mutations, notably in tumor suppressor genes (such as APC, p53) and oncogenes (like KRAS). 2) Local invasion: the tumor evolves, it infiltrates neighboring tissues, penetrating through layers such as the submucosa and muscular layers of the colon or rectum. This infiltration may extend to involve nearby lymphatic vessels. 3) Lymphatic dissemination: colorectal cancers predominantly disseminate via the lymphatic system. Lymphatic vessels serve as conduits for draining fluid and waste products from tissues, including tumor cells. Cancer cells gain access to lymphatic vessels either through direct infiltration or by shedding from the primary tumor into the surrounding tissue fluid. 4) Transport to regional lymph nodes: cancer cells are conveyed by the lymphatic fluid to regional lymph nodes. In colorectal cancer, the frequently implicated lymph nodes are those situated along the inferior mesenteric artery, superior rectal artery, and inferior mesenteric vein. 5) Adhesion and establishment: upon reaching lymph nodes, cancer cells must adhere to the lymph node endothelium and extravasate into the node parenchyma. This intricate process hinges on interactions between adhesion molecules on the surface of cancer cells and the endothelial cells within the lymph node. 6) Proliferation and expansion: Within the lymph node environment, cancer cells encounter a microenvironment conducive to their survival and proliferation. This microenvironment is shaped by various factors, including cytokines, growth factors, and interactions with immune cells. Cancer cells may undergo proliferation within the lymph node, ultimately forming micrometastases or macrometastases over time. 7) Further dissemination: following the establishment of metastases in regional lymph nodes, cancer cells possess the potential to disseminate to more distant lymph nodes or other organs via the bloodstream, a process known as hematogenous metastasis. This contributes significantly to the progression of colorectal cancer to advanced stages.

## Colorectal cancer lymph node metastasis markers

At present, the precise process and mechanism of tumor cells entering lymphatic vessels, transporting and disseminating with lymph fluid are still unclear. Recent studies have found that tumor cells first secrete corresponding chemokine receptors before lymphatic metastasis, prompting cancer cells to perform directional chemotactic movement to lymphatic vessels and attach to the surrounding wall of the vessel, and gradually at the junction of endothelial cells. Pseudopodia are protruded and eventually enter the lumen through an amoeba-like movement across the endothelial space (19). Other studies have found that in the process of rapid proliferation of the tumor to the surrounding tissue for infiltration and growth, the pressure in the cancer mass gradually increases, causing edema, and the tension of the lymphatic capillary anchor wire increases, pulling the wall and making it expand. The loose endothelial cell junctions are separated from each other and open to form inter-endothelial channels (20). At this time, a large number of cancer cells infiltrating around the tube wall invade lymphatic capillaries from the inter-endothelial channels until distant metastasis through active diffusion and push by extra-luminal pressure (21). Tumors break away from the primary site, enter the surrounding matrix, enter the lymphatic circulation system under the action of various cytokines, adhere to the endothelial cell wall, and migrate out of the lymphatic vessels to form new metastases. This process involves a complex molecular mechanism of action; many adhesion molecules, proteolytic enzymes, cytokines, and growth factors are involved in all aspects of the lymphatic system involved in tumor growth and metastasis (22, 23) (**Table 1**).

**Table 1:** Recently identified biomarkers involved in lymph node metastasis of colorectal cancer.

Biomarkers	Function in lymph node metastasis	Relevant references
*VEGF-C*	lymphangiogenesis	(24–27)
*VEGF-D*	lymphangiogenesis	(28, 29)
*VEGFR-3*	lymphangiogenesis	(30)
*IMP3*	Tumor lymphatic invasion	(31, 32)
*PRL-3*	Tumor lymphatic invasion	(33)
*TrkB*	Tumor lymphatic invasion, lymphangiogenesis	(34–38)
*HSP47*	Tumor lymphatic invasion	(39, 40)
*PROX1*	Lymphangiogenesis	(41, 42)
*IGFIR*	Tumor lymphatic invasion, lymphangiogenesis	(43)
*ESAM*	Tumor lymphatic invasion	(21, 44, 45)
*Nav1.6*	Tumor lymphatic invasion	(46)
*AQP1*	Tumor lymphatic invasion, lymphangiogenesis	(47, 48)
*AQP3*	Tumor lymphatic invasion, lymphangiogenesis	(49)
*AQP5*	Tumor lymphatic invasion, lymphangiogenesis	(50)
*CDX2*	Epithelial–mesenchymal transition (EMT)	(51)

VEGF-C, vascular endothelial growth factor C9; VEGF-D, vascular endothelial growth factor D; VEGFR-3, vascular endothelial growth factor receptor 3; IMP3, insulin-like growth factor II mRNA binding protein 3; PRL-3, phosphatase of regenerating liver-3; TrkB, tyrosine kinase receptor B; HSP47, heat shock protein 47; PROX1, prospero homeobox 1; IGFIR, insulin-like growth factor I receptor; ESAM, Embedded Secure Access Module; Nav1.6, Sodium Channel Nav1.6; AQP1, aquaporin1; AQP3, aquaporin3; AQP5, aquaporin5.

### VEGF-C, VEGF-D, and VEGFR-3

Vascular endothelial growth factor-C (VEGF-C), belonging to the VEGF family of angiogenic factors, selectively triggers VEGF receptor-3 (VEGFR-3) activation, a receptor predominantly expressed on the lymphatic endothelium (25). VEGF-C is a key regulator of lymphatic vessel formation, a process known as lymphangiogenesis. VEGF-C promotes the proliferation and branching of lymphatic vessels originating from existing ones. This heightened density of lymphatic vessels offers a conduit for cancer cells to disseminate from the primary tumor to adjacent lymph nodes and eventually to distant organs. Additionally, VEGF-C has the capability to augment the permeability of lymphatic vessels. This makes it easier for cancer cells to enter the lymphatic system from the primary tumor site (30). Once inside the lymphatic vessels, cancer cells can travel to regional lymph nodes and beyond. VEGF-C can directly promote the migration and invasion of cancer cells. It can induce changes in the tumor microenvironment that support the migration of cancer cells towards lymphatic vessels (30). Additionally, VEGF-C can enhance the ability of cancer cells to degrade the extracellular matrix, facilitating their invasion into lymphatic vessels. VEGF-C expression is often correlated with lymph node metastasis in various types of cancer. High levels of VEGF-C are associated with an increased likelihood of cancer cells spreading to regional lymph nodes. Once cancer cells reach the lymph nodes, they can continue to spread further to other organs via the lymphatic system. In summary, VEGF-C plays a multifaceted role in lymphatic metastasis by promoting lymphangiogenesis, increasing lymphatic vessel permeability, facilitating tumor cell migration and invasion, and promoting lymph node metastasis. Targeting VEGF-C and its downstream signaling pathways may therefore be a promising strategy for inhibiting lymphatic metastasis and controlling the spread of cancer.

Higher levels of VEGFC expression in CRC tumors correlate with poorer prognosis and lower survival rates. This suggests that VEGFC may serve as a prognostic marker for evaluating the aggressiveness of the disease and predicting patient outcomes. In colorectal cancer, the expression of VEGF-C in rectal cancer (75%) significantly exceeded that in normal adjacent tissue (25%). This elevated expression correlated with tumor differentiation, Dukes stage, and lymph node metastasis, while showing no significant correlation with sex or age. Moreover, VEGF-C-positive rectal cancer exhibited higher lymphatic vessel density compared to VEGF-C-negative counterparts. Additionally, lymphatic metastases demonstrated greater lymphatic vessel density compared to non-lymphatic metastases (24). VEGFC primarily acts through its receptor VEGFR-3 to induce lymphangiogenesis, the formation of new lymphatic vessels (52). In CRC, increased lymphangiogenesis facilitated by VEGFC promotes tumor cell dissemination through lymphatic vessels, leading to lymph node metastasis (53). Furthermore, VEGFC has been associated with promoting metastasis in CRC by enhancing tumor cell invasion and migration (54). It supports the spread of cancer cells from the primary tumor to distant sites, which is a critical factor in cancer progression. However, specific mutations in VEGFC itself are less commonly reported compared to its overexpression; any alterations in the signaling pathways involving VEGFC can contribute to enhanced metastatic potential. Mutations in genes that regulate VEGFC or its receptors (such as VEGFR-3) might also impact its function and contribute to cancer progression. Overall, VEGFC plays a multifaceted role in colorectal cancer, influencing both tumor growth and metastasis, and it represents a promising target for therapeutic interventions aimed at controlling disease progression.

**Figure 1:**
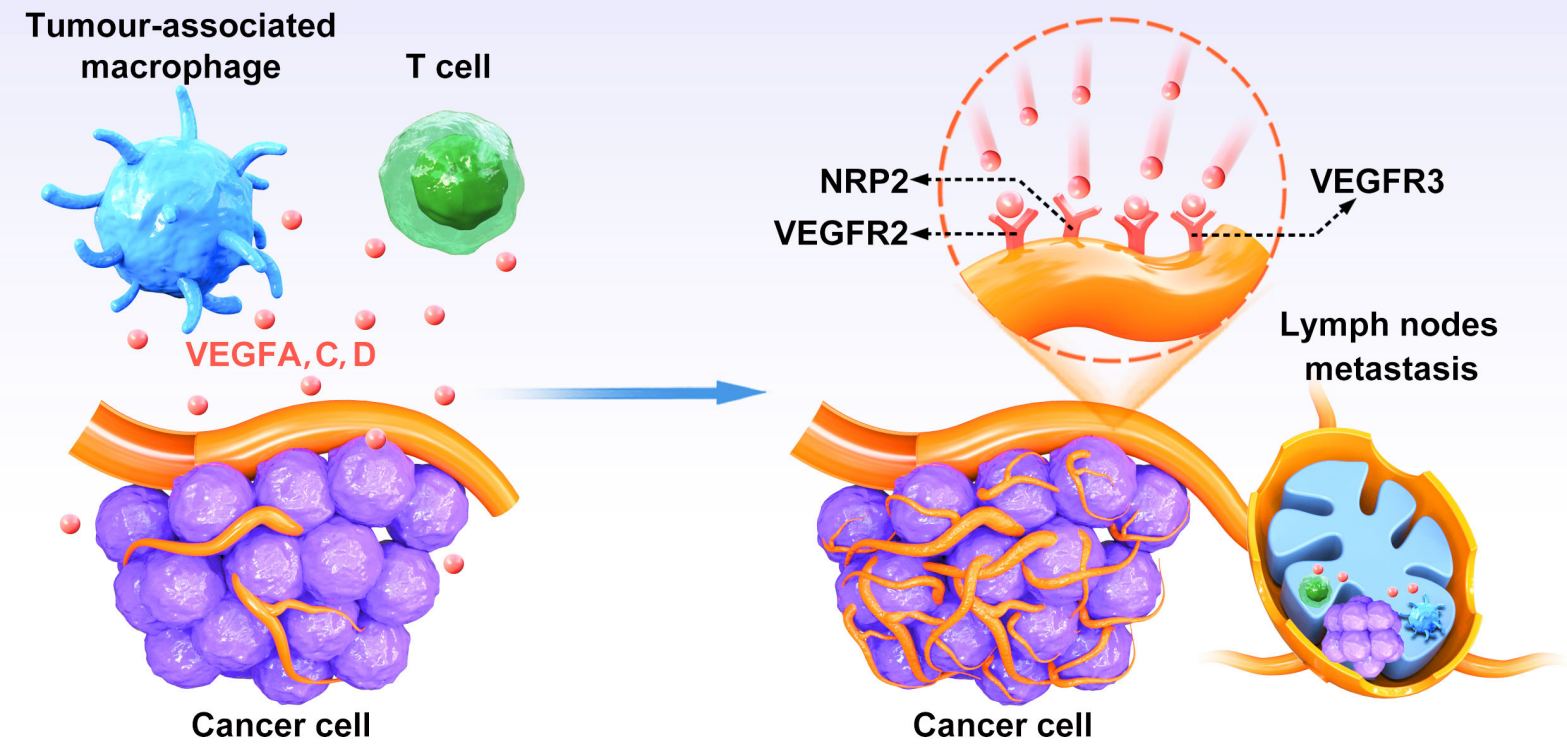
The relationship between VEGFs–VEGFR-3 pathway and lymphangiogenesis. VEGFC and VEGFD secreted by CRC cells bind to VEGFR-3 on lymphatic endothelial cells to induce activation of the P13K/AKT signaling pathway and enhance DNA replication, leading to lymphatic endothelial cells proliferation and tumor neovascularization.

VEGF-D, another VEGFR-3 ligand, has been reported as an independent prognostic marker for survival in colorectal cancer (28). Recent studies have revealed that VEGF-D also plays a significant role in promoting tumor-associated lymphangiogenesis, inducing the enlargement of draining lymphatics, and facilitating lymph node metastasis (29). Elevated expression of VEGFR-3 in colon cancer has been linked to decreased survival rates, indicating a potential axis involving VEGF-C and VEGFR-3 in colorectal cancer progression (30) (**Figure 1**). Additionally, research has demonstrated that activation of the VEGF-C/VEGFR-3 axis in lymphatic endothelial cells can promote metastasis by enhancing lymphangiogenesis both within tumors and in their surrounding environment (30). Both VEGF-C and VEGF-D are known to stimulate the proliferation, migration, and survival of endothelial cells. Specifically, the VEGF-C/VEGFR-3 signaling pathway plays a crucial role in the continued development of the lymphatic vascular network following the initial formation of lymphatic sacs, rather than solely maintaining the lymphatic vasculature in adulthood (55). These findings suggest that the expression levels of VEGF-C, VEGF-D, and VEGFR-3 could influence the prognosis of rectal cancer by influencing the formation of new lymphatic vessels (24).

VEGF-D, similar to VEGF-C, plays a critical role in promoting lymphatic vessel growth and metastasis in colorectal cancer. It enhances tumor cell invasion and migration, facilitating the spread of CRC cells to distant sites via lymphatic vessels (56). This metastatic process is crucial in the progression of CRC and can influence patient outcomes. Furthermore, elevated levels of VEGF-D in CRC tumors have been associated with poorer prognosis and reduced survival rates. Targeting VEGF-D and its signaling pathways may offer therapeutic opportunities in managing CRC. Inhibiting VEGF-D-mediated lymphangiogenesis and metastasis could potentially slow down disease progression and improve patient outcomes.

### Insulin-like growth factor II mRNA receptor 3

IMP3, also known as tumor overexpressed K homologous region-containing protein (II), is an mRNA-binding protein that can specifically bind to IGF-II mRNA during embryonic development and participate in the regulation of IcF-II mRNA localization, stability, reverse transcription, and translation; regulate the expression of IGF-II; and then participate in the regulation of tumor cell growth, differentiation, apoptosis, adhesion, and movement (57, 58). IMP3 is not expressed or weakly expressed in normal adult tissues, but it is highly expressed in various cancers and is closely related to tumor proliferation, invasion, and metastasis (57). IMP3 has been implicated in the induction of EMT, a cellular program that endows cancer cells with increased migratory and invasive capabilities (59). During EMT, cancer cells lose their epithelial characteristics and acquire mesenchymal properties, allowing them to detach from the primary tumor, invade surrounding tissues, and enter the lymphatic system (59). IMP3 may promote lymphangiogenesis, the formation of new lymphatic vessels. By enhancing the expression of lymphangiogenic factors or directly affecting lymphatic endothelial cells, IMP3 could contribute to the expansion of lymphatic networks, providing additional routes for cancer cells to disseminate to regional lymph nodes and beyond. Several studies have reported a correlation between IMP3 expression and lymph node metastasis in various cancers. High levels of IMP3 expression have been observed in lymph node metastases compared to primary tumors, suggesting a potential role for IMP3 in facilitating the metastatic spread of cancer cells through the lymphatic system (60). In colorectal cancer, IMP3 is typically overexpressed rather than mutated. This overexpression is associated with the aggressive behavior of tumors. High levels of IMP3 are often found in advanced stages of CRC and are linked to increased tumor invasiveness, metastasis, and poorer overall survival rates. Wei et al. conducted a study analyzing IMP3 expression in paired biopsy and resection specimens from 71 CRC cases, aiming to determine its correlation with various clinicopathological parameters (61). The Spearman correlation test revealed a robust linear association between IMP3 expression and both lymph node metastasis (p = 0.004) and TNM stage (p = 0.005). Patients exhibiting high-stage colorectal adenocarcinoma (CRA) and diffuse IMP3 expression exhibited significantly poorer survival rates (p < 0.0001) compared to those without diffuse IMP3 expression (p = 0.0038). Furthermore, multivariate analysis identified diffuse IMP3 expression, serosal invasion, lymph node ratio (LNR), tumor stage, and adjuvant chemotherapy as independent prognostic factors in colorectal adenocarcinoma (CRA) (31). A study has shown that IMP3 directly binds to the 3′-UTR of MEKK1 mRNA, thereby regulating its stability, promoting MEKK1 expression, and subsequently activating the MEK1/ERK signaling pathway, ultimately facilitating the malignant biological processes of colorectal cancer cells (62).

In summary, IMP3 appears to play a multifaceted role in lymphatic metastasis by promoting cancer cell behaviors conducive to metastasis, potentially influencing lymphangiogenesis, and correlating with lymph node metastasis. Further research is needed to elucidate the precise mechanisms by which IMP3 contributes to lymphatic metastasis and to explore its potential as a therapeutic target or prognostic marker in colorectal cancer patients.

### Liver regeneration phosphatase protein 3 (PRL-3)

PRL-3, also known as tyrosine kinase 4A3, is a member of the protein tyrosine phosphatase family. It is highly expressed in a variety of malignant tumors and is related to various elements of the metastasis process, including affecting cell cycle, survival, angiogenesis, adhesion, cytoskeleton remodeling, epithelial–mesenchymal transition (EMT), motility, and invasion (63). PRL-3 exhibits a multifaceted role in promoting angiogenesis via VEGF activation, enhancing extracellular matrix adhesion through Erk activation, regulating RhoGTPase to facilitate cytoskeletal remodeling, epithelial–mesenchymal transition (EMT), motility, invasion, and angiogenesis. Additionally, PRL-3 plays a role in modulating actin dynamics, microtubule organization, and intermediate filament regulation (64). PRL-3 significantly augments the migratory and invasive capacities of cancer cells by orchestrating cytoskeletal rearrangements and turnover of focal adhesions (65). These actions promote cell motility and enable invasion through extracellular matrix barriers. In the context of lymph node metastasis, heightened PRL-3 expression empowers cancer cells to breach lymphatic vessels and migrate towards regional lymph nodes. Moreover, PRL-3’s involvement in triggering EMT, a cellular reprogramming process where epithelial cells lose their polarity and cell–cell adhesion, leads to the acquisition of a mesenchymal phenotype characterized by heightened migratory and invasive capabilities. Thus, through its influence on EMT, PRL-3 facilitates the spread of cancer cells from the primary tumor site to regional lymph nodes and distant organs (66). Additionally, PRL-3 plays a pivotal role in regulating lymphangiogenesis, the formation of new lymphatic vessels. By fostering lymphangiogenesis, PRL-3 creates additional conduits for cancer cell dissemination from the primary tumor to regional lymph nodes, thus promoting lymphatic metastasis (64). Furthermore, PRL-3 exerts its metastasis-promoting effects by modulating various signaling pathways critical for cancer metastasis, including the phosphoinositide 3-kinase (PI3K)/Akt pathway, the mitogen-activated protein kinase (MAPK) pathway, and the Src family kinase signaling pathway (67–69). Activation of these pathways by PRL-3 enhances cell survival, proliferation, migration, and invasion, all pivotal processes in lymph node metastasis (70). PRL-3’s ability to regulate the activity of MMPs, enzymes crucial for degrading the extracellular matrix and facilitating tumor invasion and metastasis, further reinforces its metastatic potential (71). By augmenting MMP activity, PRL-3 aids in dismantling extracellular matrix barriers, enabling cancer cells to infiltrate surrounding tissues and access lymphatic vessels. Kazuhiko Hatate et al. conducted an immunohistochemical analysis to investigate the correlation between PRL-3 expression and clinicopathological characteristics in resected tissues from 107 colorectal cancer patients (33). Their findings revealed a significant association between PRL-3 expression and several classic prognostic factors, including the pN factor (p<0.0001), synchronous liver metastasis (SLM) (p<0.0001), pT factor (p=0.0002), preoperative CEA levels (p<0.0001), and preoperative CA19-9 levels (p<0.0001) (33). Multivariate logistic regression analysis of PRL-3 expression identified the pN factor (p<0.0001), CEA levels (p<0.0001), and CA19-9 levels (p<0.0001) as the remaining independent associations with PRL-3 expression (33). These findings suggest that the promotion of liver metastasis by PRL-3 may potentially be mediated through mechanisms involving lymph node metastasis and elevated levels of tumor markers in the serum.

The overexpression, rather than specific mutations of of PRL-3, is a critical factor in promoting colorectal cancer metastasis. The heightened expression of PRL-3 is also associated with elevated invasion of the lymphatic and venous systems, metastasis to lymph nodes and peritoneum, and an escalation in tumor stage (66). A study showed that phosphatase PRL-3 is an independent marker of aggressiveness and distant dissemination of locally advanced CRC and a useful tool to classify the accuracy of stage III CRC regarding the risk of distant relapse (72). Furthermore, PRL-3 expression in colorectal cancers may contribute to the establishment of liver metastasis, particularly at the step in which cancer cells leave the circulation to extravasate into the liver tissue. In addition, PRL-3 is expected to be a promising biomarker for identifying colorectal cancer patients at high risk for distant metastases (73).

In summary, PRL-3 emerges as a key player in lymphatic metastasis, orchestrating cancer cell migration and invasion, facilitating epithelial–mesenchymal transition (EMT), potentially modulating lymphangiogenesis, and correlating with lymph node metastasis. Comprehensive exploration of the molecular mechanisms underlying PRL-3’s involvement in lymphatic metastasis is warranted, alongside investigations into its potential utility as a therapeutic target or prognostic marker in patients with colorectal cancer.

### Tyrosine kinase receptor B

Brain-derived neurotrophic factor (BDNF) belongs to the neurotrophin (NT) family, exerting crucial roles in the development and repair of the nervous system (74). It interacts with two main receptors: tropomyosin-related receptor kinase B (TrkB), with high affinity and specificity, and the pan-NT receptor p75 (p75NTR), with lower affinity. Upon binding to TrkB, BDNF triggers autophosphorylation of tyrosine residues within the intracellular domain, initiating downstream signaling cascades such as RAS/MAPK and PI3K/AKT (75). Additionally, BDNF binds to the low-affinity receptor p75NTR, which plays various roles including the regulation of cell survival and differentiation during neuronal development. Moreover, BDNF/TrkB signaling has been implicated in tumor progression, metastasis, and chemotherapy response across several human malignancies including neuroblastoma, ovarian, head and neck, lung, and colorectal cancer (76). Koji Tanaka et al. discovered a significant association between the mRNA level of TrkB in colorectal cancer (CRC) tissues and lymph node metastasis (p = 0.022) (35, 38). In addition, TrkB can also induce the expression of VEGF-A and VEGF-C to increase and promote the increase in tumor blood vessels and lymphangiogenesis, thereby promoting tumor invasion and lymph node metastasis (34, 36, 37). Activation of TrkB signaling has been shown to promote cancer cell survival, proliferation, and resistance to apoptosis (76). This could contribute to the growth and expansion of primary tumors, facilitating the subsequent steps of metastasis, including lymphatic dissemination. TrkB signaling has been implicated in the induction of EMT, a process whereby cancer cells acquire mesenchymal properties, including increased motility and invasiveness (76). Through EMT, cancer cells can detach from the primary tumor, invade surrounding tissues, and enter the lymphatic system, facilitating metastasis. TrkB activation has been associated with increased cancer cell migration and invasion in various types of cancer (76). By promoting cytoskeletal rearrangements, cell motility, and extracellular matrix degradation, TrkB signaling may facilitate the invasion of cancer cells into lymphatic vessels and subsequent dissemination to regional lymph nodes (77). TrkB signaling can influence the tumor microenvironment by promoting angiogenesis, immune evasion, and the production of pro-metastatic factors (78). These changes in the tumor microenvironment may create a permissive niche for cancer cell dissemination and colonization within lymphatic vessels and lymph nodes.

*Correlation with lymphatic metastasis*. While TrkB’s role in lymphatic metastasis specifically is not as extensively studied as its role in other aspects of cancer progression, there is evidence to suggest that increased TrkB expression or activation correlates with lymphatic metastasis in certain types of cancer (79). Elevated TrkB levels in primary tumors have been associated with a higher incidence of lymph node involvement and poorer clinical outcomes in some studies (79, 80).

In colorectal cancer, TrkB is often overexpressed rather than mutated. This overexpression is associated with increased tumor aggressiveness, resistance to apoptosis (programmed cell death), and enhanced metastatic potential. High TrkB expression is associated with poor prognosis in CRC patients and enhanced malignant potential in terms of proliferation, migration, invasion, and anoikis inhibition in CRC cells. (81). TrkB can not only increase the anoikis resistance of colon cancer cells but also induce EMT, which play an important role in the metastasis of rectal cancer (81, 82). The work of Yu et al. suggested that overexpression of TrkB in colon cancer possibly plays roles in inhibiting apoptosis, promoting proliferation and invasion, facilitating tumor progression by lymphangiogenesis-associated metastasis (83). Furthermore, a study indicated that BDNF/TrkB signaling protects human colon cancer cells from EGFR inhibition (84). Thus, TrkB can be used as a preoperative predictor of lymph node metastasis in colorectal cancer. However, the predictive ability of molecular markers needs to be further verified.

### Heat shock protein 47

Heat shock protein 47 (HSP47) is a member of the small molecule HSP family, which can play an important role as a molecular chaperone in the process of protein folding by assembling newly synthesized proteins or reassembling misfolded proteins (85). HSP47 is located in the endoplasmic reticulum secreted by collagen, can specifically bind to procollagen, play a role in the process of endoplasmic reticulum folding, assembly and transport of procollagen, and participate in the regulatory mechanism under stress (86). HSP47 plays a crucial role in collagen maturation and secretion (86). Collagen is a major component of the ECM, and its remodeling is essential for cancer cell invasion and metastasis. HSP47-mediated regulation of collagen deposition and remodeling in the tumor microenvironment may influence cancer cell interactions with the ECM, potentially facilitating invasion into lymphatic vessel (87). HSP47-mediated alterations in the ECM composition and organization may promote tumor–stromal interactions that support cancer cell migration, invasion, and metastasis, including lymphatic dissemination (88, 89). HSP47 and HSP47-dependent collagen secretion enhanced cancer cell–platelet interaction, thereby enhancing cancer cell clustering, which is crucial for cancer cell colonization at distant sites (87). Through proteomic and immunohistochemical analyses, Koichiro Mori et al. revealed that the expression of HSP47 in tumor tissues of colorectal cancer patients and the abundance of HSP47-positive spindle cells in the tumor stroma were notably elevated compared to those in adjacent normal colon mucosa (39). Moreover, the abundance of HSP47-positive spindle cells in the tumor stroma emerges as a more informative marker for identifying lymph node metastasis.

Multivariate analysis further elucidated that spindle cells exhibiting heightened HSP47 expression levels not only act as independent markers for disease-free survival and overall survival in colorectal cancer (CRC) patients but also serve as independent predictive biomarkers for lymph node metastasis in CRC (40). Elevated HSP47 expression showed significant associations with tumor progression, including advanced T stage, lymph node metastasis, and venous invasion, and higher TNM stage (40). Hence, high HSP47 expression may serve as a novel predictive biomarker for identifying CRC patients with lymph node metastasis. Kaplan–Meier analysis revealed that patients with high HSP47 expression levels had markedly poorer overall survival compared to those with low HSP47 expression levels. Additionally, multivariate analyses highlighted HSP47 expression as an independent predictive marker for both lymph node metastasis and poor overall survival in CRC patients (40). However, HSP47 mutations are not commonly reported in colorectal cancer; any alterations in its function due to mutations could theoretically impact collagen production and ECM composition. Such changes might either enhance or impair the tumor’s ability to metastasize, depending on how the mutations affect HSP47’s chaperone activity. However, more research is needed to elucidate the specific role of HSP47 mutations in colorectal cancer.

In summary, while the precise role of HSP47 in lymphatic metastasis remains to be fully elucidated, its involvement in ECM remodeling, tumor–stromal interactions, and potentially EMT suggests that HSP47 may contribute to the metastatic process, including lymphatic dissemination. Further research is needed to better understand the mechanisms by which HSP47 influences lymphatic metastasis and to explore its potential as a therapeutic target in colorectal cancer.

### Prospero homeobox protein 1

Prospero homeobox protein 1(PROX1), a member of the homeodomain transcription factor family, is a regulatory protein involved in the growth and development of multiple organs and tissues and involved in lymphangiogenesis (42). PROX1 is highly expressed in various malignant tumors and is involved in tumor cell differentiation, proliferation, migration, apoptosis, invasion, and tumor lymph node metastasis. PROX1 is a key transcriptional regulator of lymphatic endothelial cell differentiation and identity. It controls the expression of genes involved in lymphatic endothelial cell specification, such as lymphatic vessel endothelial hyaluronan receptor 1 (LYVE-1) and podoplanin. Dysregulation of PROX1 expression or function may disrupt lymphatic endothelial cell identity and function, potentially affecting lymphatic vessel integrity and tumor cell trafficking. PROX1 is a critical regulator of lymphatic vessel development and growth. It promotes lymphangiogenesis by inducing the expression of lymphatic-specific genes and facilitating the formation of new lymphatic vessels from pre-existing ones. In the context of cancer, increased PROX1 expression in tumor-associated lymphatic vessels has been associated with enhanced lymphangiogenesis and lymphatic vessel density, which could provide routes for cancer cell dissemination and lymphatic metastasis. Beyond its effects on lymphatic endothelial cells, PROX1 may also influence the behavior of tumor cells. Studies have suggested that PROX1 expression in cancer cells can modulate their invasive potential and metastatic propensity. In some contexts, PROX1 expression in cancer cells has been associated with increased invasiveness and lymphatic metastasis. However, the precise mechanisms by which PROX1 regulates tumor cell behavior in the context of lymphatic metastasis require further investigation. PROX1 was found to be overexpressed in 43% of colon cancer tissues, and its upregulation correlated with the downregulation of E-cadherin, advanced tumor staging, and the presence of lymph node metastasis (90).

In colon cancer, PROX1 suppresses the expression of E-cadherin at the transcriptional level through the inhibition of microRNA-9-2. This mechanism promotes epithelial–mesenchymal transition (EMT), and invasion and metastasis (91). Knockdown of PROX1 in colon cancer cell lines can reduce the expression of VEGF-A and increase the expression of angiostatin, while overexpression of PROX1 can promote tumor cell angiogenesis and lymphangiogenesis, and tumor cell proliferation and migration (92). Abdelrahman et al. compared the protein expression of PROX1 in lymph node stage II and III colorectal cancer tissues by immunohistochemistry and found that PROX1 expression level was correlated with tumor grade, lymph node metastasis, and advanced tumor stage (93).

In summary, PROX1 plays a multifaceted role in lymphatic metastasis by regulating lymphatic endothelial cell identity, promoting lymphangiogenesis, potentially influencing tumor cell behavior, and correlating with clinical outcomes. Further research is needed to fully elucidate the mechanisms by which PROX1 contributes to lymphatic metastasis and to explore its potential as a therapeutic target or prognostic marker in colorectal cancer.

### Insulin-like growth factor I receptor

The insulin-like growth factor 1 (IGF-1) receptor is a transmembrane receptor belonging to the class of tyrosine kinase receptors. It becomes activated upon binding with the hormone insulin-like growth factor 1 (IGF-1) and its closely related hormone, IGF-2. This receptor serves as a mediator for the biological effects of IGF-1, a polypeptide protein hormone that shares molecular similarities with insulin. IGF1R signaling has been extensively implicated as a pivotal factor in cancer cell proliferation, survival, migration, and resistance to anticancer therapies. Therefore, targeting IGF signaling represents an appealing therapeutic strategy (94). Mounting evidence underscores that the IGF axis not only fuels tumorigenesis but also bestows resistance to standard treatments. Activation of IGF-1R signaling promotes cancer cell proliferation and survival by stimulating cell cycle progression and inhibiting apoptosis. Enhanced cancer cell proliferation may contribute to tumor growth and expansion, facilitating subsequent steps of metastasis, including lymphatic dissemination (43). IGF-1R activation has been also associated with increased cancer cell migration and invasion in various cancer types. By promoting cytoskeletal rearrangements, cell motility, and extracellular matrix degradation, IGF-1R signaling may facilitate the invasion of cancer cells into lymphatic vessels and subsequent dissemination to regional lymph nodes. Elevation of IGF-IR signaling can enable cancer cells to evade anoikis by suppressing the activation of p53 and p21 (95). IGF-IR also facilitates anchorage-independent growth via RACK1-mediated activation of STAT3 and Akt (96). Moreover, IGF-IR/Akt signaling induces the expression of LIP, which is an isoform of CCAAT enhancer binding protein-β known to suppress anoikis (97). Inhibiting IGF-IR increases the vulnerability of cancer cells to anoikis, diminishes circulating tumor cells in the bloodstream, and suppresses cancer metastasis (98). IGF-1R signaling has been implicated in the induction of EMT, a cellular program that endows epithelial cells with mesenchymal-like properties associated with increased motility and invasiveness. Through EMT, cancer cells may acquire the ability to detach from the primary tumor, invade surrounding tissues, and enter the lymphatic system, facilitating lymphatic metastasis (99). While direct evidence linking IGF-1R to lymphangiogenesis is limited, IGF-1R signaling has been implicated in promoting angiogenesis, the formation of new blood vessels. Crosstalk between blood and lymphatic vessels in the tumor microenvironment suggests that factors promoting angiogenesis may indirectly influence lymphangiogenesis, potentially affecting lymphatic metastasis. Deficiency of IGF1R in the lung tumor microenvironment (TME) impedes tumor initiation and progression by reducing inflammation and mitigating immunosuppression within lung tumors (94).

In colorectal cancer tissues, significantly higher rates of expression were observed for insulin-like growth factor I receptor (IGFIR) (46%), VEGF (53%), and VEGF-C (46%) compared to normal and colorectal adenoma tissues. Furthermore, elevated IGFIR expression, which regulates VEGF and VEGF-C, has been identified as a predictor for lymph node metastasis in human colorectal cancer (100). Stimulation by IGF-1 causes proliferation *in vitro* while blocking IGF1-R inhibits growth of colorectal cancer cells (101). The work of Li et al. suggested that elevated IGF-1 and IGF-1R expression in CRC tissues was correlated with lymph node metastasis and tumor TNM stage (102).

In summary, IGFs are frequently elevated in colorectal cancer (CRC), promoting excessive autocrine and paracrine stimulation of cell growth. This process often involves overexpression or increased activation/accessibility of IGF receptors, facilitating the transmission of IGF-related signals. Various molecules and biochemical mechanisms modulate these processes, significantly influencing the outcome of IGF-stimulated pathways and potentially leading to neoplastic transformation when the balance is disrupted beyond repair. Targeting IGF-1R signaling pathways could represent a potential therapeutic strategy to inhibit lymphatic metastasis and improve patient outcomes in cancer.

### Endothelial-specific adhesion molecule and Endoglin

Endothelial-specific adhesion molecule (ESAM), an endothelial tight junction protein specifically expressed at endothelial tight junctions and on platelets (103). Endoglin (ENG) is a transmembrane glycoprotein expressed on endothelial cells, serving as a co-receptor for multiple ligands of the transforming growth factor beta (TGF-β) family (45). The expression of ESAM was found to be elevated in hypervascular metastatic tumor tissues compared to normal tissues across various tumors. Cell culture investigations revealed that conditioned medium from B16F10 melanoma cells heightened ESAM expression in endothelial cells, leading to enhanced endothelial migration and tube formation (44). The endothelial migration and tube formation induced by B16F10 medium were notably reduced upon downregulation of ESAM through siRNA transfection (44). Both ESAM and Endoglin exhibited upregulation within the lymphatics of colorectal cancer (CRC) (21). ESAM is involved in the regulation of endothelial cell junctions, which are critical for maintaining vascular integrity. By modulating adherens junctions and tight junctions between endothelial cells, ESAM influences vascular permeability and barrier function. Changes in vascular permeability may affect cancer cell intravasation into lymphatic vessels during metastasis (44). While primarily studied in the context of blood vessel formation (angiogenesis), ESAM expression has been implicated in endothelial cell sprouting and vessel formation (104). Tumor-associated lymphatic vessels are often structurally abnormal and hyperpermeable, and factors influencing angiogenesis may indirectly affect lymphangiogenesis, potentially contributing to lymphatic metastasis. While primarily studied in the context of immune cell recruitment to sites of inflammation, alterations in immune cell trafficking within the tumor microenvironment may impact cancer progression and metastasis, including lymphatic dissemination. Furthermore, mutations in ESAM may disrupt its normal function, leading to increased cancer cell mobility and invasive behavior. Such mutations might influence the cancer’s ability to spread from the primary site to other parts of the body.

In summary, while the specific role of ESAM in lymphatic metastasis remains to be fully elucidated, its involvement in endothelial cell junction regulation, angiogenesis, immune cell trafficking, and its association with cancer progression suggest that ESAM may play a role in the metastatic process. Further research is needed to better understand the mechanisms underlying ESAM’s contribution to lymphatic metastasis and to explore its potential as a therapeutic target or prognostic marker in colorectal cancer.

### Voltage-gated sodium channels

Voltage-gated sodium channels (VGSCs) are macromolecular protein complexes embedded in the cell membrane, consisting of a pore-forming α subunit and one or more associated smaller β subunits. The voltage-gated sodium channel (VGSC) alpha subunit family consists of nine members, namely, Nav1.1 to Nav1.9, which are encoded by the SCN1A to SCN11A genes. The voltage-gated sodium channel (VGSC) is prominently expressed in excitable cells, including neurons and cardiomyocytes, where it plays a pivotal role in generating action potentials and facilitating the transmission of neural signals (105). Nevertheless, studies have revealed the expression of VGSCs in tumor cells across a spectrum of cancers, including breast cancer, cervical cancer, colon cancer, melanoma, neuroblastoma, non-small cell lung cancer, ovarian cancer, and prostate cancer[9]. VGSC activity has been associated with increased cancer cell migration and invasion in various cancer types (106). VGSCs contribute to the regulation of cell motility through their involvement in the control of cytoskeletal dynamics and cell adhesion (107). VGSC activity can influence intracellular ion concentrations and pH dynamics in cancer cells. Dysregulation of ion homeostasis and pH dynamics is associated with cancer cell survival, proliferation, and invasiveness (107). Changes in ion fluxes mediated by VGSCs may create an environment conducive to cancer cell migration and invasion, potentially facilitating lymphatic metastasis. VGSC activity has been implicated in the regulation of ECM remodeling, including the degradation of ECM proteins by matrix metalloproteinases (MMPs). ECM remodeling is critical for cancer cell invasion and metastasis, including lymphatic dissemination. VGSC-mediated regulation of MMP activity may influence cancer cell invasion into lymphatic vessels and subsequent metastatic spread (108). However, specific studies focusing on VGSCs and lymphatic metastasis are limited, increased expression or activity of VGSCs has been observed in various cancers, and their dysregulation has been associated with tumor progression and metastasis in some studies. Therefore, it is plausible that VGSCs may contribute to lymphatic metastasis by promoting cancer cell migration, invasion, and ECM remodeling (106).

Studies have shown that elevated expression of specific VGSC isoforms, such as Nav1.5 and Nav1.7, correlates with poor prognosis in colorectal cancer patients. This suggests that VGSCs could serve as potential biomarkers or therapeutic targets (109). Studies have indicated that Nav1.5 upregulates the expression of CRC-inducible genes via the MAPK signaling pathway, consequently fostering metastasis in colon cancer (110). Lin et al. discovered that overexpression of Nav1.1 and Nav1.6, rather than Nav1.5, exhibited a positive correlation with lymph node metastasis in CRC. Subsequent immunohistochemistry analyses revealed low expression levels of Nav1.5 and Nav1.6 in non-metastatic lymph nodes, while Nav1.6 displayed high expression in metastatic lymph nodes in colorectal cancer (46). These findings suggest that elevated expression of Nav1.1 and Nav1.6 may facilitate lymph node metastasis in colorectal cancer (46).

In summary, while further research is needed to fully elucidate the role of VGSCs in lymphatic metastasis, existing evidence suggests that VGSC activity may influence various aspects of the metastatic process, including cancer cell migration, invasion, and ECM remodeling. Targeting VGSCs or their downstream signaling pathways could represent a potential therapeutic strategy to inhibit lymphatic metastasis and improve patient outcomes in cancer.

### Aquaporin channels

Aquaporins (AQPs) are transmembrane channels found expressed in a diverse array of cells and tissues across the mammalian body (48). Aquaporins are tetrameric transmembrane proteins composed of six alpha helices that allow cells to maintain water balance through the osmotic gradient of the cell membrane (48). To date, 13 distinct aquaporins (AQPs), encoded by the genes AQP0 through AQP12, have been identified. Among them, AQP1, AQP2, AQP4, AQP5, and AQP8 function as classic aquaporins, facilitating the transport of water and small solutes across cell membranes. Conversely, AQP3, AQP6, AQP7, and AQP9 are permeable to both glycerol and water (111). AQPs are expressed not only in cancer cells but also in stromal cells within the tumor microenvironment, including endothelial cells and immune cells. Alterations in aquaporin (AQP) expression or function within stromal cells can significantly influence the tumor microenvironment, thereby affecting cancer cell behavior and metastatic propensity, particularly in relation to lymphatic metastasis. AQPs have been implicated in the modulation of cell migration and invasion in cancer. Although the precise mechanisms governing this regulation remain incompletely understood, AQPs are thought to exert their effects on cell motility through modulation of cell volume regulation, cytoskeletal dynamics, and interactions with the extracellular matrix (ECM). Changes in AQP expression or function may therefore affect cancer cell migration and invasion into lymphatic vessels. Lymphangiogenesis, the formation of new lymphatic vessels, is a critical process in cancer metastasis to the lymph nodes and beyond. While the specific role of AQPs in lymphangiogenesis is not well characterized, AQPs may indirectly influence lymphatic vessel formation by regulating the hydration and volume of lymphatic endothelial cells or by modulating signaling pathways involved in lymphangiogenesis.

Several studies have highlighted the significant roles played by aquaporins (AQPs) in colorectal cancer cell proliferation, migration, invasion, and angiogenesis (112). Reports have shown altered expression of various aquaporin isoforms in CRC compared to normal colorectal tissue. For example, increased expression of AQP1, AQP3, AQP4, and AQP5 has been reported in CRC tissues, while AQP8 expression may be downregulated. The expression intensity of AQP1, AQP3, and AQP5 are associated with the differentiation, lymph node metastasis, and distant metastasis of colon cancer (47, 49, 50). hEGF upregulates AQP3 expression through the PI3K/AKT pathway (113). These changes in aquaporin expression levels suggest potential roles for these channels in CRC development and progression.

In summary, while the precise involvement of AQPs in lymphatic metastasis necessitates further investigation, current evidence indicates that AQPs could impact several facets of the metastatic cascade. These include tumor cell hydration, alterations in the microenvironment, and cell migration, invasion, and potentially lymphangiogenesis. Further research is essential to elucidate the mechanisms through which AQPs contribute to lymphatic metastasis and to assess their potential as therapeutic targets or prognostic markers in colorectal cancer.

### Caudal type homeobox 2

Caudal type homeobox 2 (CDX2) serves as a pivotal transcription factor orchestrating crucial roles in intestinal development and differentiation. Its presence in colorectal cancer, where it acts as a hallmark of intestinal differentiation, underscores its significance (51). However, while CDX2’s involvement in tumor progression is well-established, its precise implication in lymph node metastasis remains a subject of active investigation. Here is a refined overview of CDX2’s potential role in lymph node metastasis. 1) Association with tumor differentiation: CDX2 expression typically aligns with well-differentiated colorectal tumors, often indicating a less aggressive phenotype with lower metastatic tendencies. Conversely, studies suggest that the diminishment of CDX2 expression could correlate with tumor dedifferentiation and heightened aggressiveness, potentially elevating the risk of lymph node metastasis (114). 2) Regulation of epithelial–mesenchymal transition (EMT): emerging evidence implicates CDX2 in governing genes integral to epithelial–mesenchymal transition (EMT), a process pivotal in fostering tumor invasion and metastasis. Diminished CDX2 expression might disrupt EMT-related pathways, fostering an invasive phenotype conducive to lymph node colonization and metastasis (51). 3) Interaction with Wnt signaling pathway: CDX2 operates downstream of the Wnt signaling pathway, frequently dysregulated in colorectal cancer. Aberrations in Wnt signaling are implicated in tumor initiation, progression, and metastasis. CDX2’s influence on Wnt signaling components could substantially shape tumor behavior, potentially impacting metastatic dissemination, including lymph node involvement (115). 4) Regulation of cell adhesion and migration: CDX2 exerts regulatory control over genes governing crucial aspects of cell adhesion, migration, and invasion. Loss of CDX2 expression may disrupt these regulatory mechanisms, fostering tumor cell detachment from the primary site and facilitating their migration towards lymphatic vessels, thereby augmenting the likelihood of lymph node metastasis (116).

Study showed that loss of CDX2 expression in primary tumors and lymph node metastases is specific for mismatch repair deficiency in colorectal cancer (117). Furthermore, enhanced CDX2 promoter methylation is associated with gene silencing in a subgroup of colorectal cancer patients with lymph node metastasis and shorter survival times (118). Overexpression of CDX2 resulted in reduced MMP-2 expression, leading to diminished cell proliferation, invasion, and migration in human colon cancer cell lines. Conversely, knockdown of CDX2 enhanced MMP-2 expression and increased these cellular processes. Notably, CDX2 overexpression suppressed EMT markers, highlighting its role in inhibiting colorectal cancer cell viability, invasion, and metastasis (51). Furthermore, mutations in the CDX2 gene are highly uncommon in CRC—only 3 of 224 previously sequenced colorectal cancer cell lines and tumors harbor a mutation (0.9% mutant allele frequency), and all of those mutations occur in repeat sites of cancers with microsatellite instability (119). However, the prognostic significance of CDX2 expression in the context of lymph node metastasis remains a contentious topic, necessitating further investigation to elucidate its precise role in this aspect of colorectal cancer progression.

## Mechanisms of lymphatic metastasis in colorectal cancer

Cancer metastasis appears to unfold through a series of events wherein organ selectivity by tumor cells is greatly influenced by physical factors such as blood and lymphatic flows, and biological molecules present within the tumor microenvironment. As outlined earlier, emerging evidence indicates that lymph node (LN) metastasis not only offers prognostic insights but also actively contributes to the development of distant metastases in vital organs, ultimately leading to lethality. Yet, our understanding of the molecular mechanisms underlying this process remains limited. To date, several sets of chemokines and their receptors have been suggested to play pivotal roles in lymph node metastasis.

*Invasion of tumor cells into lymphatic vessels.* The initial step in lymphatic metastasis involves the invasion of CRC cells from the primary tumor into nearby lymphatic vessels. This process can be facilitated by various factors, including the loss of cell adhesion molecules, increased motility and invasiveness of cancer cells, and the degradation of the extracellular matrix (ECM) by matrix metalloproteinases (MMPs) and other proteases.

*Lymphangiogenesis*. Lymphatic vessels within and surrounding the primary tumor undergo lymphangiogenesis, the formation of new lymphatic vessels. This process is stimulated by factors secreted by tumor cells, such as vascular endothelial growth factors (VEGFs) and other lymphangiogenic factors. The newly formed lymphatic vessels provide routes for CRC cells to enter the lymphatic system.

*Intraluminal tumor cell migration*. Once inside the lymphatic vessels, CRC cells migrate through the vessel lumen in a process facilitated by their ability to survive in the lymphatic fluid and evade immune surveillance. Cancer cells may interact with lymphatic endothelial cells and utilize various adhesion molecules and signaling pathways to promote their migration within the lymphatic vessels.

*Transport to regional lymph nodes*. CRC cells transported by lymphatic vessels can reach regional lymph nodes, which are the primary sites of metastasis in many cases of CRC. The lymphatic flow, driven by factors such as lymphatic contractility and tumor-induced lymphangiogenesis, plays a crucial role in the transport of cancer cells to regional lymph nodes. Additionally, factors secreted by tumor cells, such as chemokines and growth factors, may attract cancer cells to lymph nodes.

*Colonization and growth in lymph nodes*. Once CRC cells reach regional lymph nodes, they may extravasate from the lymphatic vessels and establish micrometastases within the lymph node parenchyma. Factors promoting cell survival, proliferation, and angiogenesis within the lymph node microenvironment contribute to the growth and expansion of metastatic deposits in lymph nodes.

*Further dissemination*. Metastatic CRC cells within lymph nodes may undergo further dissemination to distant sites through the bloodstream or additional lymphatic vessels, contributing to distant metastasis and disease progression.

**Figure 2:**
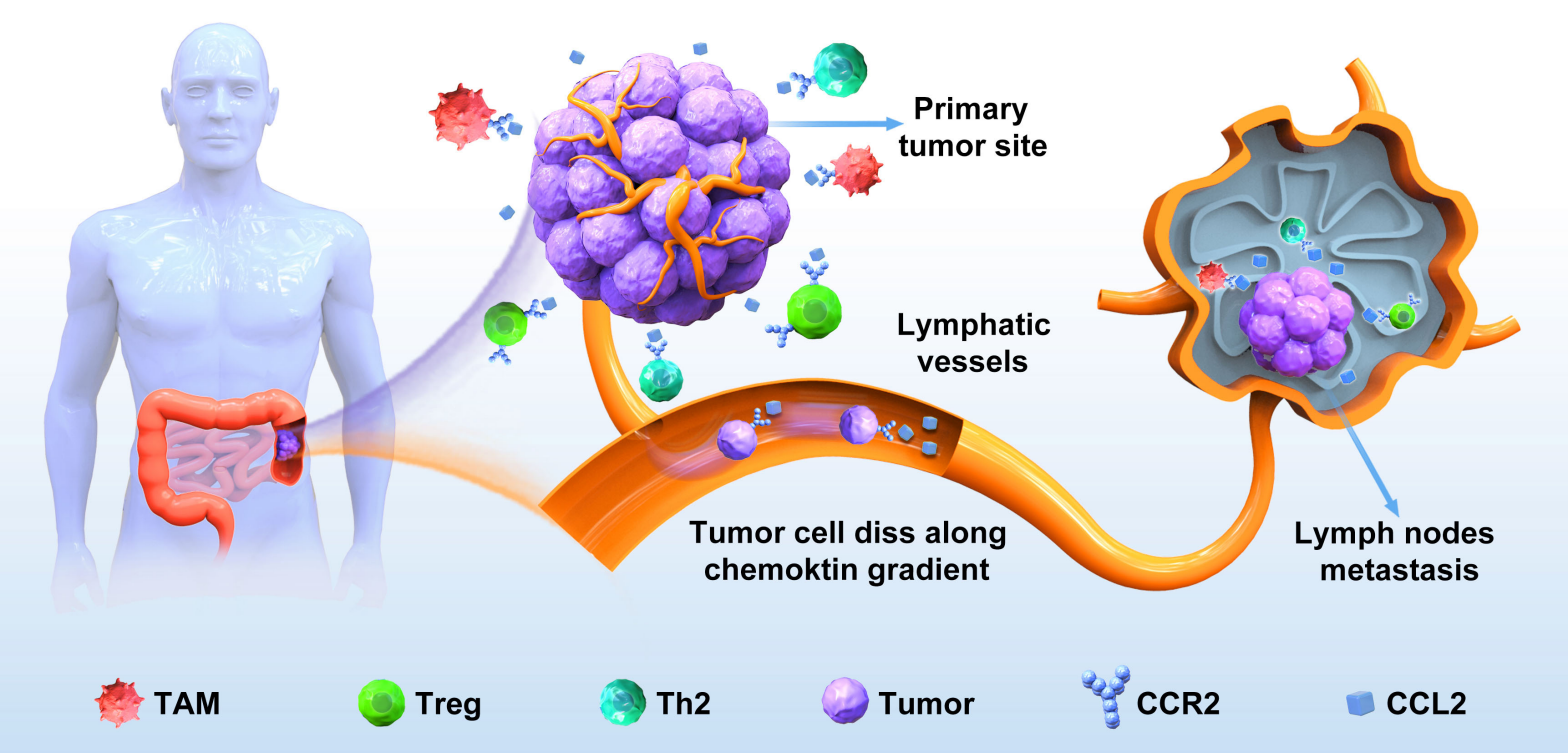
The CCL2‐CCR2 axis in the lymph node metastasis of colorectal cancer. CRC cells detach from the primary site, enter the surrounding stroma, and secrete a large amount of CCL2, which binds to CCR2 on the surface of immune cells such as TAM in the tumor immune microenvironment, so that invasive colorectal cancer cells are driven by the CCL2–CCR2 axis to invade lymphatic vessels, adhere to endothelial cell walls, and migrate to lymph nodes to form new metastases.

One of the well-studied examples is the CCL2–CCR2 axis in lymph node metastasis of oral squamous cellcarcinoma (120). CCL2, also recognized as monocyte chemoattractant protein-1 (MCP-1), stands as the first extensively studied CC chemokine, exhibiting a preferential affinity for its receptor CCR2. Earlier investigations have revealed the involvement of the CCL2–CCR2 signaling axis in fostering pathological angiogenesis, promoting the survival and invasion of tumor cells and facilitating the recruitment of immune inhibitory cells. In colorectal cancer, CCL2 has been observed to accelerate CRC cell proliferation and invasion. Moreover, the use of a specific CCR2 antagonist has shown promise in suppressing NF‐κB activation during DOX‐induced senescence, thereby effectively blocking CCL2 transcription (121). The CCL2–CCR2 signaling axis plays a crucial role in various stages of tumorigenesis. This axis supports tumor cell growth and proliferation at the primary tumor site. Furthermore, as malignant cells disseminate from their primary locations to initiate metastasis, the CCL2–CCR2 axis can stimulate cancer cells to invade surrounding tissues, enter the circulatory system, and migrate along specific chemotactic gradients to establish metastatic colonies at distant sites. Study has shown that in oral squamous cell carcinoma, the CCR2–CCL2 axis is directly involved in lymph node metastasis (120). This study used an orthotopic lymph node metastasis model in mice and found that tumor-associated macrophages (TAMs) infiltrated into lymph node sinuses secreted a large amount of CCL-2 in an autocrine or paracrine manner to induce CCR-2-positive tumor cells metastasize to lymph nodes (120). In metastatic colorectal cancer, a significant infiltration of tumor-associated macrophages (TAMs) occurs in the liver, facilitating the growth of metastatic cancer cells through TAM-mediated immunosuppression. Intriguingly, the CCL2–CCR2 axis not only promotes tumor progression by recruiting and reprogramming TAMs but also supports cancer invasion and metastasis through the secretion of CCL2 by TAMs (**Figure 2**). This reciprocal crosstalk and mutual promotion create a positive feedback loop, fostering the induction and activation of TAMs within the tumor microenvironment (TME). Consequently, these interactions establish favorable conditions conducive to tumor growth and metastasis.

## Conclusion

Lymphatic invasion and lymph node metastasis in colorectal cancer represent a key time point in the occurrence of colorectal cancer and are key prognostic factors in patients with colorectal cancer. According to the staging of lymph node metastasis, it is important to develop an appropriate treatment plan to prevent the recurrence of colorectal cancer and improve the prognosis. While current lymph node metastasis markers have provided valuable insights into colorectal cancer progression and prognosis, they also come with several limitations when considering them as therapeutic targets: Many current lymph node metastasis markers lack the necessary specificity and sensitivity to accurately predict metastatic spread or effectively target metastatic lesions. This can result in false positives or negatives, leading to misdiagnosis or ineffective treatments. For example, metastasis is a multifaceted, dynamic process involving intricate interactions between cancer cells and the microenvironment. Existing markers may fail to capture the dynamic changes occurring during metastatic progression, limiting their utility as therapeutic targets. Furthermore, cancer cells often develop resistance to targeted therapies, leading to treatment resistance and disease progression. Targeting a single marker may not suffice to counter the diverse resistance mechanisms employed by cancer cells to evade therapeutic interventions. In addition, targeting a specific marker implicated in lymph node metastasis may inadvertently affect normal tissues or disrupt other physiological processes, resulting in off-target effects and unintended consequences for patient health.

Overall, while current lymph node metastasis markers of CRC have provided valuable insights into cancer biology, their limitations underscore the need for continued research to identify novel therapeutic targets and develop more effective strategies for preventing or treating metastatic disease. Combination therapies targeting multiple aspects of metastasis and personalized treatment approaches based on individual tumor profiles may hold promise for improving CRC patient outcomes in the future.
